# Noninvasive 3D-CT simulation versus glue injection to localize small pulmonary nodules prior to anatomical segmentectomy: a randomized controlled trial

**DOI:** 10.1093/icvts/ivad156

**Published:** 2023-09-19

**Authors:** Linhai Fu, Wenbin Wu, Alisherjon Oblokulov, Ting Zhu, Zhifeng Ma, Haiyong Wang, Yuanlin Wu, Zhupeng Li, Guangmao Yu, Chu Zhang, Miao Zhang

**Affiliations:** Department of Thoracic Surgery, Shaoxing People’s Hospital, Shaoxing, China; Department of Cardiothoracic Surgery, Xuzhou Central Hospital, Xuzhou, China; Department of Thoracic Surgery, Shaoxing People’s Hospital, Shaoxing, China; Department of Thoracic Surgery, Shaoxing People’s Hospital, Shaoxing, China; Department of Thoracic Surgery, Shaoxing People’s Hospital, Shaoxing, China; Department of Thoracic Surgery, Shaoxing People’s Hospital, Shaoxing, China; Department of Thoracic Surgery, Shaoxing People’s Hospital, Shaoxing, China; Department of Thoracic Surgery, Shaoxing People’s Hospital, Shaoxing, China; Department of Thoracic Surgery, Shaoxing People’s Hospital, Shaoxing, China; Department of Thoracic Surgery, Shaoxing People’s Hospital, Shaoxing, China; Department of Cardiothoracic Surgery, Xuzhou Central Hospital, Xuzhou, China

**Keywords:** Segmentectomy, Uniportal/single-port, Video-assisted thoracoscopic surgery, Three-dimensional computed tomography bronchography and angiography, Noninvasive localization, Ground-glass opacity

## Abstract

**OBJECTIVES:**

This study aimed to investigate whether adding glue injection to three-dimensional computed tomography bronchography and angiography (3D-CTBA) has extra benefits to facilitate anatomical segmentectomy for pulmonary nodules.

**METHODS:**

We conducted a randomized controlled trial. The patients undergoing thoracoscopic segmentectomy assisted with 3D-CTBA simulation were enrolled. Then, they were divided into the 3D-CTBA group and the glue-labelling group who received additional computed tomography-guided percutaneous glue (2-octyl cyanoacrylate) injection to label the nodules. The primary outcome was the resection rate of the nodules, and the secondary measures included the operation time, complications and thorax drainage.

**RESULTS:**

A total of 173 patients were randomized into the 3D-CTBA group (89 patients) and glue-labelling group (84 patients) between January 2018 and March 2019. Before the segmentectomy, the patients using glue labelling recorded 5 (6.0%) cases of pneumothorax, 2 (2.4%) cases of haemothorax and 1 (1.2%) case of severe chest pain. All the surgical procedure was performed fluently and safely. The resection rate of the nodules was 100% in both groups. Furthermore, these patients demonstrated similar operation time [(141.5 ± 41.9) vs (142.1 ± 38.9) min], estimated blood loss [(111.3 ± 74.0) vs (106.0 ± 63.8) ml], duration of chest tube duration [(5.1 ± 3.0) vs (5.0 ± 3.5) days] and total drainage volume [(872.3 ± 643.1) vs (826.7 ± 806.0) ml], with a *P*-value of >0.05 respectively. In addition, 6 (7.1%) patients in the glue-labelling group and 6 (6.7%) patients in the 3D-CTBA group reported air leakage (>5 days) and chylothorax.

**CONCLUSIONS:**

Noninvasive 3D-CTBA alone is probably sufficient to facilitate anatomical segmentectomy. The additional invasive glue labelling could be avoided in selected patients who undergo intentional segmentectomy.

**Clinical trial registration:**

The trial was registered under the Chinese Clinical Trial Registry (ChiCTR). Identifier: ChiCTR1800018293, https://www.chictr.org.cn/showproj.html?proj=29345.

**Figure:**
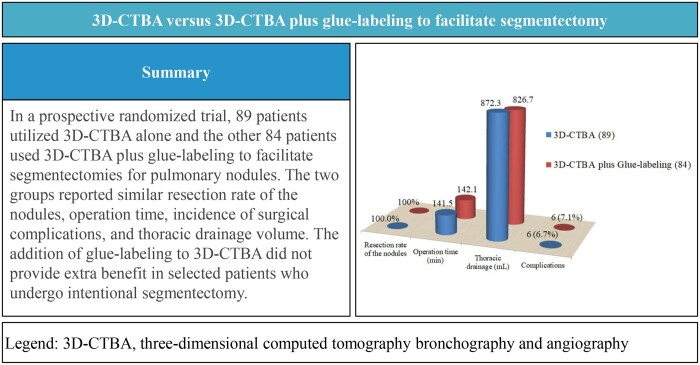

## INTRODUCTION

Pulmonary nodules have been reported to be detected on approximately 30% of the chest computed tomography (CT) images [1]. The management options of the nodules include surveillance, biopsy and surgery. The absence of preoperative localization of the pulmonary nodules may lead to inaccurate resection. However, it is difficult to palpate the ground-glass opacity (GGO) and the deep-located nodules during the operation. There are various preoperative labelling methods to locate the GGOs, such as CT-guided percutaneous insertion of a hookwire, microcoil and methylene blue. A meta-analysis found that the successful targeting rates for hook-wire, microcoil and lipiodol localization were 0.98, 0.98 and 0.99, respectively [2]. Meanwhile, the incidence of pneumothorax following hook-wire, microcoil and lipiodol localization was 0.35, 0.16 and 0.31, followed by a haemorrhage rate of 0.16, 0.06 and 0.12, respectively [2]. Our previous study investigated the efficacy of preoperative localization of small pulmonary nodules by CT-guided ZT medical glue (2-octyl cyanoacrylate) injection, and the results confirmed that glue labelling was a reliable procedure for sublobar resection [3]. Three-dimensional (3D) visualization of the anatomy of the segmental divisions is essential for a precise segmentectomy. A meta-analysis showed that 3D lung surgery simulation could significantly decrease the blood loss, operative time, incidence of conversion and complications compared with non-3D procedures [4].

To date, randomized controlled trials (RCTs) regarding the efficacy of 3D-CT bronchography and angiography (3D-CTBA) versus CT-guided percutaneous invasive glue labelling to localize the pulmonary nodules are lacking. We carried out a prospective RCT to determine the safety and reliability of the noninvasive 3D-CTBA with or without the invasive glue labelling during video-assisted thoracoscopic surgery (VATS). The implantation of the glue truly facilitated the identification of the nodules, but the procedure of a standard anatomical segmentectomy could not be simplified by the glue. Therefore, we supposed that glue labelling was non-essential for intentional segmentectomy assisted with 3D-CTBA simulation.

## PATIENTS AND METHODS

### Ethical statement

The study was approved on 28 July 2017 by the Clinical Research Ethics Committee of Shaoxing People's Hospital (Approved trial No. 2017-068). The trial was conducted under the supervision by the Data and Safety Monitoring Committee of Zhejiang University School of Medicine. All study participants provided informed consent prior to the trial. The data were presented anonymously for patients’ privacy concern.

### Study design and participants

We hypothesized that glue labelling plus 3D-CTBA was superior to 3D-CTBA alone to facilitate precise anatomical segmentectomy, including a higher resection rate of the nodules and shorter operation time because of the quicker exploration of the nodules under glue guidance.

**Figure 1:**
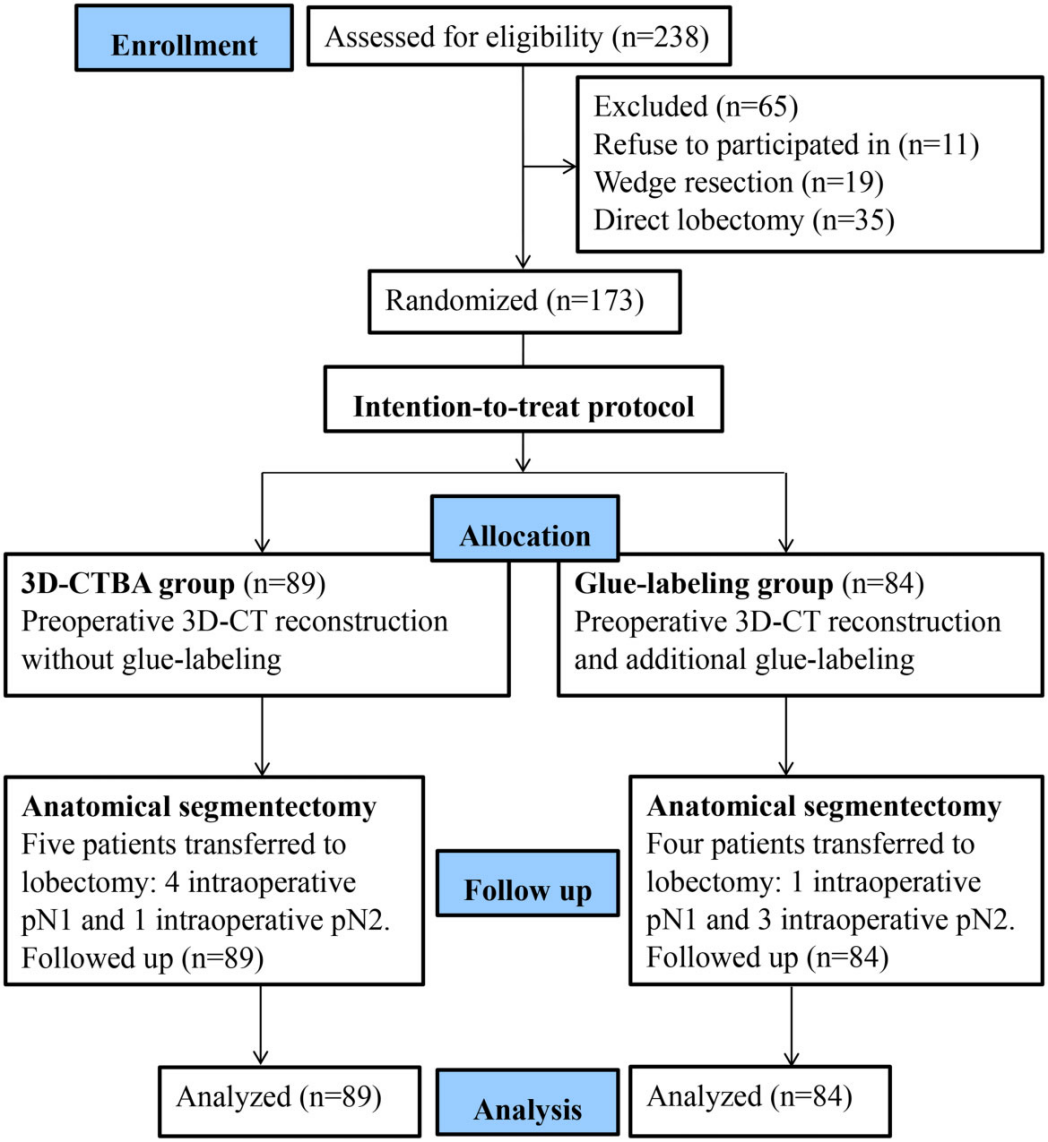
Flow chart of the trial.

This study was a prospective RCT with a parallel group design. In theory, at least 385 patients were needed for this RCT to achieve a confidence level of 95% that the real value was within ±5% of the measured value, as the type I error was set at two-sided 0.05 according to a reported statistical guidance [5]. Patients with pulmonary nodules who were scheduled to receive VATS segmentectomy between January 2018 and March 2019 at Shaoxing People's Hospital were enrolled. If the eligibility criteria were met, the patients were informed of the potential risks and benefits of the noninvasive and invasive localization procedures. Preoperative 3D-CTBA using thin-slice CT data was applied to confirm the location of the nodules and the variations of the bronchopulmonary segmental anatomy individually. Besides the 3D-CTBA, the patients in the glue-labelling group received additional CT-guided injection of medical glue (2-octyl cyanoacrylate) into the surface of the target segment adjacent to the nodule. The flowchart is illustrated in Fig. 1. This trial had been registered formally, and the identifier was ChiCTR1800018293.

### Inclusion and exclusion criteria

The patients with pulmonary nodules, suspicious of malignancy, were referred to assess the eligibility of VATS segmentectomy. The inclusion criteria were as follows: (i) persistent (during a follow-up of ≥3 months) pulmonary nodules with a maximum diameter of ≤20 mm on CT revealing the features of malignancy, or probably benign nodule ≤30 mm; (ii) no evidence of mediastinal lymph nodes involvement or distant metastases; (iii) preoperative cardiopulmonary function could tolerate pulmonary segmentectomy including a forced expiratory volume in 1 s >0.6 l and a maximum voluntary ventilation >35% of predicted; (iv) peripheral-type nodule and the deepest part of the lesion was at least 2 cm away from the major vessels for a safe margin; (v) frozen-section diagnosis of the regional lymph node was pathological negative (pN0); and (vi) aged ≥18 years and mentally competent to give written informed consent. The exclusion criteria were as follows: (i) contraindication of lung resection including compromised cardiopulmonary function with a forced expiratory volume in 1 s of ≤0.6 l and a maximum voluntary ventilation of ≤35% of predicted; (ii) the nodule was suspicious of malignancy with a diameter of >20 mm; (iii) deeply located central-type tumour with or without distant metastases; (iv) frozen-section showed tumour-positive of the lymph node; (v) previous ipsilateral thoracotomy; (vi) deep lesions with a tumour-to-intersegmental fissure distance of >2 cm which could not be precisely located by medical glue injection or accompanied with the obvious risk of bleeding.

### Randomization

The project leader produced the numbers by an online software (Research Randomizer Version 4.0 at https://www.randomizer.org), and the patients were numbered in the order of their enrolment time, matching random numbers sequentially. The randomization list was kept confidential to the patients, and the block randomized design was used for this study. The patients were not masked to treatment allocation. Then, the eligible individuals were assigned to the 3D-CTBA group and glue-labelling group, respectively.

### Preoperative three-dimensional computed tomography bronchography and angiography reconstruction and resection simulation

**Figure 2:**
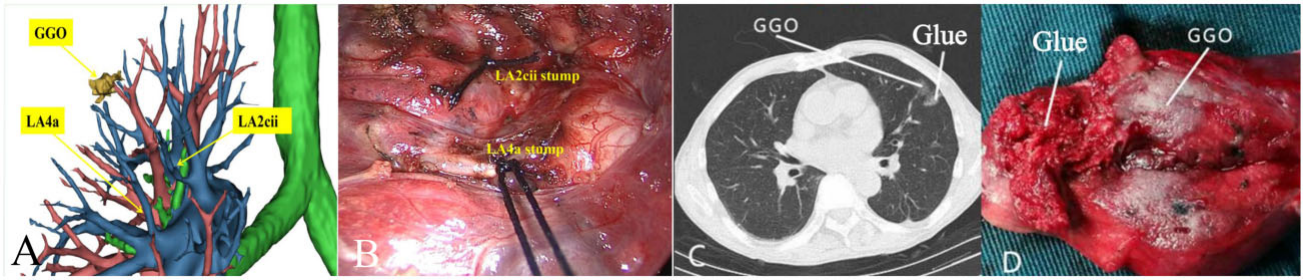
Localization of the ground-glass opacity (GGO) by three-dimensional computed tomography or glue injection labeling. (**A**) Three-dimensional computed tomography indicated a GGO in the left upper lobe located between the apical-posterior segment (LS1 + 2) and the superior lingular segment (LS4). (**B**) Intraoperative confirmation of the resection of LS2cii + 4a. (**C**) Preoperative computed tomography-guided percutaneous glue injection onto the target segment. (**D**) The resected specimen showed the removal of the GGO along with the glue.

The digital imaging and communications in medicine data of the sections (1.0- to 1.25-mm thickness) of each patient were collected, and then, the 3D-CTBA was established using DeepInsight or Mimics. The pulmonary segmental vessels were then indicated (Fig. 2A), followed by the surgical planning.

### Preoperative glue injection

Before the surgery, CT-guided percutaneous glue injection was performed under local anaesthesia using 2% lidocaine (5 ml for each patient) in the glue-labelling group to further locate the pulmonary nodules. This procedure was finished in the Department of Radiology separately [3]. Then the patients were transferred to the operation room waiting for anatomical lung resection.

### Thoracoscopic segmentectomy under the guidance of three-dimensional computed tomography bronchography and angiography

General anaesthesia and one-lung ventilation through double-lumen endobronchial tubes were used for each patient during the operation. The manipulation VATS port (3–3.5 cm) was on the 4th or 5th intercostal space of the anterior axillary line. If the pulmonary nodule could not be excised under thoracoscope due to diffuse pleural adhesion, or potential risk of major bleeding due to the complex variations of the target segment, another 1∼2 ports (1.5∼2 cm) were utilized in the 7th intercostal space of the midaxillary line. With the guidance of the 3D-CTBA, the bronchus, artery and the intrasegmental vein of the target segment could be dissected separately. The collapsed lung was re‐expanded completely with controlled airway pressure ≤20 cm H_2_O, with the bronchus of the operation side open to atmosphere while continuing ventilation of the contralateral lung. Five to twelve minutes later, an irregular demarcation developed between the inflated target segment and the deflated surrounding segments. The inflation–deflation method could identify the intersegmental plane for resection using endostaplers (Endo GIA II, United States Surgical, Norwalk, Conn; Echelon Endostapler, Ethicon Endo-Surgery, Cincinnati, Ohio). Lymph nodal sampling was routinely performed. The nodules and the glue were removed completely.

### Outcomes

The primary outcome was the proportion of R0 resection with pathological diagnosis of the nodules. The secondary indicators included the operation time, estimated blood loss, the incidence of complications and postoperative hospital stay.

### Data collection

The patients’ characteristics were known to the surgeons and the anaesthesiologists. The perioperative data collection and analysis were performed by 3 independent assessors who were unaware of the nature of the study. Two members were responsible for the collection of the raw data independently, and the other one finished the statistics of the data.

### Statistical analysis

All analyses were performed based on the intention-to-treat principle. Statistical analysis was performed using Statistical Package for the Social Sciences software version 23.0 for Windows (IBM-SPSS Inc., Armonk, NY, USA). Shapiro–Wilk test was used to investigate whether the data were normally or non-normally distributed. Normally distributed continuous data were presented as means ± standard deviations and analysed quantitatively by one-way analysis of variance. Wilcoxon test was used to compare non-normally distributed continuous data which were described using the median (interquartile range 25th–75th percentiles). The Chi-square and Fisher exact tests were used for comparing 2 or more groups of categorical variables presented as counts and percentages according to the sample size. In detail, the Fisher exact test was used when the sum of the counts in a 2 × 2 table was smaller than 40, or a cell had an expected value of <1. A two-tailed *P*-value of <0.05 was considered statistically significant.

Preliminary statistics would be performed after 40 and 80 cases of practice in each group, respectively. If the initial results failed to demonstrate the extra benefit of glue labelling for intentional segmentectomy versus 3D-CTBA alone, the trial would be stopped temporarily. Meanwhile, if the incidence of glue-related harmful events was higher than 10%, this trial was also considered to be suspended.

## RESULTS

### General information of the patients

The interim data analysis of the first 40 and 60 cases of practice showed that glue labelling did not provide additional benefit in segmentectomy versus 3D-CTBA alone, considering the similar nodule resection rate and operation time. Therefore, this study was stopped before we could reach the ideal sample size. The premature termination agreement was approved by the Ethics Committee and the Monitoring Committee involved in this trial.

Initially, 238 individuals met the inclusion criteria. Among them, 11 patients refused to participate in the trial, 35 patients needed to perform lobectomy directly because the irregular nodules were located deeply and the time spent for segmental resection couldn’t be obtained separately. Therefore, these 35 patients were excluded. The patients who underwent further lobectomy for N1/N2 disease after initial segmentectomy were included. Meanwhile, another 19 patients received wedge resection. Finally, a total of 173 patients were assigned into the 3D-CTBA group (89 patients) and the glue-labelling group (84 patients). No significant difference was observed in body mass index and pathological staging between the 2 groups, except the distribution of the pulmonary nodules (Table 1).

**Table 1:** Baseline characteristics of the patients

Characteristics	Total (*n* = 173)	3D-CTBA group (*n* = 89)	Glue-labelling group (*n* = 84)	*P*-Value
Age (years), mean (SD)	61.3 (10.5)	62.4 (9.6)	60.2 (11.3)	0.163
Gender, *n* (%)				0.444
Female	108 (62.4)	58 (65.2)	50 (59.5)	
Male	65 (37.6)	31 (34.8)	34 (40.5)	
Smoking history, *n* (%)				0.910
Current	31 (17.9)	16 (18.0)	15 (17.8)	
Quit	9 (5.2)	4 (4.5)	5 (6.0)	
Never smoked	133 (76.9)	69 (77.5)	64 (76.2)	
Pack-year of smoking, mean (SD)	37.7 (22.5)	36.3 (21.3)	39.2 (24.2)	0.685
Preoperative serum CRP^a^ (mg/l), mean (SD)	5.0 (16.7)	5.0 (14.4)	5.0 (19.0)	0.977
Body mass index (kg/m^2^), mean (SD)	23.9 (2.8)	24.0 (2.9)	23.7 (2.6)	0.383
Symptoms on admission, *n* (%)				0.303
Asymptomatic	143 (82.7)	71 (79.8)	72 (85.7)	
Cough/chest pain or stuffiness	30 (17.3)	18 (20.2)	12 (14.3)	
Localization of the nodule, *n* (%)				**0.027**
Right upper lobe	94 (54.3)	49 (55.1)	45 (53.6)	
Right middle lobe	2 (1.2)	1 (1.1)	1 (1.2)	
Right lower lobe	18 (10.4)	3 (3.3)	15 (17.9)	
Left upper lobe	48 (27.7)	29 (32.6)	19 (22.6)	
Left lower lobe	11 (6.4)	7 (7.9)	4 (4.8)	
Diameter of the nodules (mm), mean (SD)	12.7 (3.6)	13.1 (3.6)	12.3 (3.5)	0.146
Imaging type of the nodules, *n* (%)				0.346
Pure GGO	39 (22.5)	24 (27.0)	15 (17.9)	
Mixed GGO	86 (49.7)	41 (46.0)	45 (53.6)	
Solid	48 (27.7)	24 (27.0)	24 (28.5)	
Pathological diagnosis, *n* (%)				0.554
Benign	6 (3.5)	2 (2.4)	4 (4.8)	
AAH or AIS	8 (4.6)	5 (5.6)	3 (3.6)	
MIA, IA, or SCC	159 (91.9)	82 (92.1)	77 (91.6)	
Lymph node metastasis, *n* (%)				0.252
N0	164 (94.8)	84 (94.4)	80 (95.2)	
N1	5 (2.9)	4 (4.5)	1 (1.2)	
N2	4 (2.3)	1 (1.1)	3 (3.6)	
Staging of the lung cancer,^b^*n* (%)				0.429
I	150 (94.3)	77 (93.9)	73 (94.8)	
II	5 (3.1)	4 (4.9)	1 (1.3)	
III	4 (2.5)	1 (1.2)	3 (3.9)	

3D-CTBA: three-dimensional computed tomography bronchography and angiography; AAH: atypical adenomatous hyperplasia; AIS: adenocarcinoma in situ; CRP: C-reactive protein; GGO: ground-glass opacity; IA: invasive adenocarcinoma; MIA: minimally invasive adenocarcinoma; SCC: squamous cell carcinoma; SD: standard deviation.

The localization of the pulmonary nodules in different lobes was significantly different between the two groups are represented in Bold.

a The normal range of serum C-reactive protein is ≤8 mg/l.

b According to the 8th edition of the Union for International Cancer Control (UICC)/ American Joint Committee on Cancer (AJCC) tumor-node-metastasis (TNM) staging system for lung cancer.

### Surgical parameters

All the surgical procedures were performed fluently and safely, and the R0 resection rate of the target nodules was 100% with sufficient surgical margins in both groups. The bronchioles and vessels of the target segments were identified accurately during the surgery the same as the 3D-CT simulation (Fig. 2B). In a total of 173 cases, the time required for a distinct demarcation to develop between the target and the surrounding segments was 5∼12 min (median, 8 min). The glue was removed with the target segments. During the surgery, the glue on the surface of the lung was quickly identified (Fig. 2C).

No mortality was observed. The frozen-section revealed 5 cases of pN1 and 4 cases of pN2, and these patients underwent further lobectomy; however, the time spent for the previous segmentectomy was recorded separately as per the intention-to-treat protocol.

The patients in the 3D-CTBA and the glue-labelling groups demonstrated similar operation time [(141.5 ± 41.9) vs (142.1 ± 38.9) min], blood loss [(111.3 ± 74.0) vs (106.0 ± 63.8) ml], stations [(3.1 ± 2.6) vs (3.0 ± 2.2)] and number [(7.3 ± 7.0) vs (6.8 ± 5.7)] of resected lymph nodes, duration of postoperative chest tube drainage [(5.1 ± 3.0) vs (5.0 ± 3.5) days], total drainage volume [(872.3 ± 643.1) vs (826.7 ± 806.0) ml] and postoperative hospital stay [(6.4 ± 3.1) vs (6.3 ± 3.6) days], with a *P*-value of >0.05, respectively (Table 2).

**Table 2:** Perioperative parameters of the patients

Variables	Total (*n* = 173)	3D-CTBA group (*n* = 89)	Glue-labelling group (*n* = 84)	*P*-Value
Surgical procedure of VATS, *n* (%)				0.097
Uniportal	106 (61.3)	56 (62.9)	41 (48.8)	
Two-port	65 (37.6)	32 (36.0)	39 (46.4)	
Three-port	2 (1.2)	1 (1.1)	4 (4.8)	
Range of resection, *n* (%)				**0.039**
Single segmentectomy	144 (83.2)	69 (77.5)	75 (89.3)	
Combined or extended segmentectomy	29 (16.8)	20 (22.5)	9 (10.7)	
Labelling-related complications, *n* (%)	–	–	8 (9.5)	–
Severe chest pain	–	–	1 (1.2)	
Pneumothorax	–	–	5 (6.0)	
Haemothorax	–	–	2 (2.4)	
Lymph node resection, *n* (%)				0.608
Dissection	40 (23.1)	18 (20.2)	22 (26.2)	
Sampling	121 (69.9)	64 (71.9)	57 (67.9)	
Omitting	12 (6.9)	7 (7.9)	5 (5.9)	
Lymph node harvested				
Stations, mean (SD)	3.0 (2.4)	3.1 (2.6)	3.0 (2.2)	0.715
Numbers, mean (SD)	7.1 (6.4)	7.3 (7.0)	6.8 (5.7)	0.662
Operation time (min), mean (SD)	141.8 (40.4)	141.5 (41.9)	142.1 (38.9)	0.928
Blood loss (ml), mean (SD)	108.7 (69.1)	111.3 (74.0)	106.0 (63.8)	0.609
Chest tube drainage				
Duration (days), mean (SD)	5.0 (3.2)	5.1 (3.0)	5.0 (3.5)	0.729
Total volume (ml), mean (SD)	850.2 (725.0)	872.3 (643.1)	826.7 (806.0)	0.681
Postoperative complications, *n* (%)	12 (6.9)	6 (6.7)	6 (7.1)	0.916
Chylothorax, *n*	4 (2.3)	1 (1.1)	3 (3.6)	
Air leak >5 days, *n*	8 (4.6)	5 (5.6)	3 (3.6)	
Serum CRP on POD2 (mg/l), mean (SD)	102.3 (46.9)	107.2 (45.0)	97.1 (48.5)	0.158
Postoperative hospital stay (days), mean (SD)	6.3 (3.3)	6.4 (3.1)	6.3 (3.6)	0.885

3D-CTBA: three-dimensional computed tomography bronchography and angiography; CRP: C-reactive protein; POD, postoperative day; SD: standard deviation; VATS: video-assisted thoracoscopic surgery.

The range of segmental resection was significantly different between the two groups are represented in Bold.

However, more combined segmentectomy was performed in the 3D-CTBA group. The incidence of complications was similar in both groups. Six patients (6.7%) in the 3D-CTBA group and 6 (7.1%) in the glue-labelling group reported complications, including 8 cases of air leakage (>5 days) and 4 cases of chylothorax. Chylothorax was confirmed by the presence of chylomicrons in the pleural effusion. Finally, the removal of the nodules and the glue was confirmed by intraoperative finding (Fig. 2D). The pathological diagnoses exhibited 6 cases of inflammation, 3 cases of atypical adenomatous hyperplasia, 5 cases of adenocarcinoma in situ, 18 cases of minimally invasive adenocarcinoma, 135 cases of invasive adenocarcinoma and 6 cases of squamous cell carcinoma (Table 1).

### Findings associated with glue injection

For the 84 patients in the glue-labelling group, the duration for CT-guided glue injection was (12.7 ± 3.8) min. The additional cost of glue injection was estimated to be ¥2100 for each patient. Moreover, the interval between glue labelling and surgical resection was (5.4 ± 4.1) h. Emergent pain killer was used for 1 patient (1.2%) because of the severe chest pain after glue injection. Meanwhile, they reported 5 (6.0%) cases of pneumothorax and 2 (2.4%) cases of haemothorax, which were alleviated timely by conservative procedures including oxygen inhalation by nasal tube. Haemostasis was performed successfully for the 2 patients with haemothorax, and the estimated extra bleeding before segmentectomy was 70 and 150 ml, respectively.

### Follow-up

All the patients enrolled in this study were followed up after the segmentectomy by telephone or internet for (50.7 ± 3.4) months (until 1 January 2023), and they reported satisfactory quality of life. For lung cancer patients who were diagnosed with lung cancer after curative-intent therapy, low-dose chest CT and cranial magnetic resonance imaging were taken regularly for at least 5 years postoperatively. No local or distant recurrence of the tumour was recorded in this cohort.

## DISCUSSION

The present trial verified the reliability of noninvasive localization of the pulmonary nodules using 3D-CTBA simulation prior to anatomical segmentectomy. In theory, glue labelling is a reliable choice to facilitate wedge resection, and it could obviously lessen the time needed during the exploration of the pulmonary nodules. Initially, the addition of glue implementation to 3D-CTBA before segmental resection was supposed to shorten the operation time, because the nodules could be identified quickly. However, the invasive glue injection could be avoided for intentional segmentectomies as shown in this study, as the 2 groups reported similar resection rate of the nodules, operation time, and incidence of surgical complications, as well as postoperative hospital stay. Several issues need to be elucidated accordingly.

It's often not easy to identify sub-centimetric and sub-solid lesions during VATS, but there are concerns about potentially fatal air embolism during CT-guided insertion of a hookwire or microcoil. Moreover, the hookwire technique showed a relatively higher failure rate due to the wire dislodgement. The radio-guided surgery is reported to be a preferable method because of its high accuracy and low complications versus hook-wire, spiral-wire and microcoil [6]. Specifically, the widely-used CT-guided microcoil implantation can guide precise resection of the invisible and impalpable pulmonary nodules. For non-mechanical localization materials, the success rate of CT-guided medical glue and methylene blue dye localization for pulmonary nodules is 99.5% along with 4.6% of mild pneumothorax, 2.0% of mild haemothorax, 0.3% of haemoptysis, pleural reaction and pain [7]. Preoperative CT-guided localization of the small indeterminate pulmonary nodules using patent blue vital dye is feasible; whereas the incidence of asymptomatic pneumothorax is 29.4% [8]. Furthermore, the mechanical labelling procedures should be restricted to diminish the potential risk of tumour dissemination during the implantation of the labelling tools.

The interactive pulmonary 3D-CTBA models for surgery simulation and planning increase the surgeon confidence in recognizing anatomical structures [9]. Moreover, segmentectomy with a safety proximal distance (>2.0 cm) could be feasible for inner small-sized (<2.0 cm) non-small-cell lung cancer (NSCLC) [10]. Considering the complexity of vascular as well as the bronchial variations, and the difficulty in nodule localization during segmental resection, 3D-CTBA facilitates the safe and simplified anatomical sub-lobectomy and reduces the complications obviously [11]. Our study revealed that 3D-CTBA might be sufficient for precise and fluent segmentectomies for small-sized pulmonary nodules. The additional invasive glue labelling accompanied with certain complications could be avoided for the patients who undergo intentional segmentectomy, because both groups revealed similar R0 resection rate and operation time. Actually, preoperative noninvasive localization of the pulmonary nodules by 3D-CTBA facilitates the fluent performance of VATS complex segmentectomy. The additional invasive glue labelling could be avoided in selected patients who undergo intentional segmentectomy. However, considering the extra benefit during the identification of small GGOs versus 3D-CTBA alone, glue labelling is a reliable choice for wedge resection.

Moreover, the 3D-CTBA could be utilized to determine the intersegmental cutting plane during segmentectomy [12]. To date, the methods for identifying the intersegmental plane during segmentectomy include inflation–deflation technique, selective resected segmental inflation, indocyanine green, endobronchial dye, 3D-CTBA and virtual-assisted lung mapping [13]. Moreover, the arterial-ligation-only method can accurately and inexpensively identify the intersegmental plane, which is especially suitable when veins or bronchi are difficult to resect [14]. However, a one-size-fits-all method is not feasible because each method has its pros and cons.

The quality control of segmentectomy is another important issue. To date, there are limited RCTs regarding segmentectomy versus lobectomy for small-sized NSCLC [15]. Continued improvement in quality control during anatomical segmentectomy is essential for NSCLC patients to achieve oncological outcomes comparable to lobectomy. The margin distance of >10 mm or margin-to-tumour ratio ≥1 should be recommended for stage I NSCLC during sublobar resection, as larger margin provided a favourable prognosis for patients with solid-predominant type or non-lepidic adenocarcinoma [16]. Thoracoscopic anatomical single or combined basal segmentectomy is technically challenging because of the variation and deep location of the vessels and bronchi. The complications following 3D-CTBA-guided complex sub-lobectomy are reported to be decreased along with the accumulated surgical experience [17]. Besides anatomical partial lobectomy, single-direction basal segmentectomy could be performed fluently via the inferior-pulmonary-ligament approach or an interlobar-fissure approach [18].

### Limitations

The present study had obvious limitations. First, the 3D-CTBA models may deviate from the intraoperative findings. Second, when the frozen section was performed after the removal of the target segment, there were 9 specimens reported benign lesion, and a wedge resection rather than a segmentectomy was sufficient. However, a precise wedge resection of the impalpable nodule without labelling was sometimes hard or even impossible. Third, the sample size of this trial was quite small as the study was stopped prematurely before we could reach the ideal sample size. Another limitation was that the single-blind design of the trial was in fact hard to achieve, because both the surgeons and the patients realized the CT-guided injection of the medical glue. If the patients did not receive the invasive glue labelling, they actually knew that they were divided into the 3D-CTBA group. As a result, the subjective bias and selection bias between the groups were unavoidable, and the evidence quality of this RCT was obviously jeopardized. Further high-volume and well-designed studies are warranted to verify our findings.

## CONCLUSION

Noninvasive 3D-CTBA simulation alone is probably sufficient to facilitate a precise segmentectomy. The additional invasive glue labelling could be avoided in selected patients who undergo 3D-CT-guided intentional segmentectomy.

## Data Availability

The original data analysed during the current study are available from the corresponding author on reasonable request. The authors are accountable for all aspects of the work in ensuring that questions related to the accuracy or integrity of any part of the work are investigated and resolved.

## ABBREVIATIONS

3D: Three-dimensional
3D-CTBA: Three-dimensional computed tomography bronchography and angiography
GGO: Ground-glass opacity
NSCLC: Non-small-cell lung cancer
RCT: Randomized controlled trial
VATS: Video-assisted thoracoscopic surgery
